# Feasibility and user experience of augmented reality psychoeducation and mindfulness body scan for chronic low back pain

**DOI:** 10.3389/fpain.2025.1600637

**Published:** 2025-07-01

**Authors:** Robin Conen, Nikolai Hepke, Jörg Lohscheller, Steffen Mueller, Ana N. Tibubos

**Affiliations:** ^1^ Department of Diagnostics in Healthcare & eHealth, Trier University, Trier, Germany; ^2^ Department of Computer Science, Trier University of Applied Sciences, Trier, Germany; ^3^ Department of Computer Science/Therapeutic Sciences, Trier University of Applied Sciences, Trier, Germany; ^4^ Department of Psychosomatic Medicine and Psychotherapy, University Medical Center Mainz, Mainz, Germany

**Keywords:** augmented reality, chronic low back pain, pain education, mindfulness body scan, user experience, feasibility

## Abstract

**Background:**

Chronic low back pain (CLBP) is prevalent and a multimodal therapy is indicated, including psychological treatment. Effective conventional treatments involve psychoeducation and mindfulness-based body scans, while virtual reality offers superior but temporary pain relief. Augmented Reality (AR), which combines conventional and virtual methods, is a novel therapeutic strategy.

**Methods:**

We investigated the viability and acceptability of an AR intervention for CLBP by incorporating psychoeducation and mindfulness-based body scan techniques. 40 participants in two studies with a one-arm design underwent an educational AR intervention (Study I, *n*_1_ = 18) and an enhanced version with an additional body scan (Study II, *n*_2_ = 22). The studies focused on evaluating technical feasibility and multiple facets of user experience.

**Results:**

The results demonstrated high feasibility with low dropout rates (Study I: 10%, Study II: 0%). User experience ratings ranged from “Above Average” to “Excellent,” with the advanced intervention receiving higher ratings. While Study I showed no significant changes in affect pre- vs. post-intervention, Study II exhibited a significant reduction in negative affect and improved valence. Qualitative analysis provided insights into technical requirements and user perceptions.

**Discussion:**

The AR prototype emerges as a promising psychoeducational tool for CLBP, aligning with current treatment guidelines and providing a basis for future controlled clinical trials. Limitations include the absence of a high-pain intervention group, as Study I reported a pain intensity of *M* = 1.05 and Study II reported *M* = 1.77 (Range: 0–10). Further research such as clinical trials with control groups is required to validate the efficacy of the piloted approach. The AR-based psychoeducation and mindfulness body scan intervention for CLBP demonstrated technical feasibility and a good user experience.

**Clinical Trial Registration:**

Open Science Framework.io; https://doi.org/10.17605/OSF.IO/DSW5X and https://doi.org/10.17605/OSF.IO/XVJBZ

## 1 Introduction

Worldwide, low back pain (LBP) affects 60%–80% of the adult population, with 10% progressing to chronic conditions (CLBP) (1). Among these chronic cases, 85% are categorized as chronic non-specific low back pain, lacking a clear cause (2). Given limited effectiveness and potential side effects of medication-based treatments, there is a growing demand for non-pharmacological alternatives (3) to enhance treatment outcomes and develop effective behavioral interventions (4). Current treatment guidelines recommend behavioral changes, physical activity, psychoeducation (5), and physiotherapy targeting at strengthening and stabilisation trunk muscles (6, 7) to alleviate pain and improve function. Educational intervention encompasses targeted strategies within an educational framework, designed to achieve specific objectives by utilizing resources informed by educational knowledge and the education system (8), without a primary focus on mental health. Psychoeducation, on the other hand, is a distinct therapeutic approach that integrates educational methods with cognitive behavioral therapy to impart knowledge about mental illness to patients (9). In the context of CLBP, the objective of psychoeducation is to elucidate the biopsychosocial interactions contributing to pain development, educate on risk factors such as catastrophizing, avoidance, and negative beliefs about pain, and to advocate for self-management strategies (10). Psychoeducational interventions improve disease understanding and promote coping mechanisms, physical activity, quality of life, and symptom management (11, 12). Psychoeducation strengthens self-efficacy and interrrupts the fear-pain cycle (13, 14). An additional treatment approach is mindfulness-based stress reduction (MBSR), particularly the body scan technique, which can reduce negative emotions, pain, anxiety, and pain perception in older adults with CLBP (15).

With the advancement of healthcare digitalization, novel non-pharmacological interventions, such as virtual reality treatments, has been developed, demonstrating superior effects compared to conventional therapies in the treatment of CLBP (16). Immersive technologies are categorized along the reality-virtuality continuum described by Milgram and Kishino (17). These technologies encompass visual displays ranging from real to virtual environments, including Augmented Reality (AR) and Virtual Reality (VR) (18–20). AR enables simultaneous interaction between digital and physical elements in real-time. Conversely, VR offers full immersion in virtual realities and represents the extremes of this continuum (21, 22). VR was found to shift attention away from pain to more pleasant visual, tactile, and auditory stimuli, thereby reducing pain intensity, catastrophizing, and associated psychological symptoms in CLBP patients (19, 23, 24). A meta-analysis found that VR training may mitigate kinesiophobia and pain intensity in CLBP (25). Initial psychoeducational VR training programs demonstrated a game-based approach to interactive knowledge transfer, albeit with only short-term pain relief (26), as well as the feasibility of immersive VR programs for pain neuroscience education (54). Thus, immersion and user focus in VR can support mindfulness and enhance pain management (27). 360-degree nature scenes promoted relaxation techniques (28) and illustrated the benefits of immersion in CLBP. A recent review article on VR for the treatment of CLBP treatment presented substantial evidence supporting its safety and tolerability (60). However, it highlighted methodological limitations and the predominance of short-term effects. This review recommends further research on safety, acceptance, and satisfaction, including targeted investigations of the risks of VR-induced spinal pain.

While VR has demonstrated positive outcomes in alleviating CLBP symptoms, AR remains underexplored despite its potential additional benefits. AR overlays virtual elements into the physical world, allowing seamless coexistence and real-time interaction, while reducing issues such as cybersickness and visual discomfort (29). Overall, AR enhances the incorporation of the real environment and body, strengthens embodiment, and provides a more authentic experience (30).

Despite these findings and advancements, a recent scoping review identified a significant research gap regarding the efficacy and feasibility of AR-based interventions for CLBP, particularly those integrating conventional and VR approaches (31). Grounded in the Unified Theory of Acceptance and Use of Technology by Venkatesh et al. (32), the review offers theory-driven recommendations for designing AR interventions for psychoeducation and MBSR-based body scans relaxation training. Thus, study aimed to assess the feasibility, acceptability, and potential benefits of AR in psychoeducational and mindfulness-based interventions for CLBP, bridging the research gap and laying the groundwork for clinical trials. The feasibility study focused on individuals with CLBP undergoing an psychoeducational AR intervention (Study I) or an enhanced version incorporating a body scan (Study II), assessing technical feasibility and facets of user experience. We formulated two hypotheses (H): An AR intervention for CLBP is technically feasible with an attrition rate of less than 50% (H1), in particular psychoeducation (H1a) and psychoeducation in combination with a MBSR body scan (H1b). Furthermore, we expect a positive user experience in AR (H2) both psychoeducation (H2a) and its combination with a MBSR body scan (H2b).

## 2 Materials and methods

### 2.1 Study design and study procedure

Two one-arm feasibility studies, with a pre-post design, investigated two scenarios: Study I (Psychoeducation) and Study II (Psychoeducation + Body Scan). The research was collaboratively designed and conducted by Trier University and Trier University of Applied Sciences and was carried out in the VR-AR laboratories of Trier University. Participants were informed that the aim of the feasibility study was to assess the technical implementation of an AR intervention for CLBP as a foundation for a subsequent clinical trial. Sociodemographic data (age, sex, height, weight, psychoeducation, marital status, and duration of back pain) and pain intensity data were collected before the intervention using questionnaires. In Study I, questionnaires were administered in AR environment. Due to hand-tracking issues with Microsoft HoloLens 2 ® and subsequent prolonged completion times, paper-pencil questionnaires were administered in Study II. Both studies evaluated the (a) dropout rate and (b) user experience. For the feasibility evaluation a dropout rate of <50% considered a feasibility success. User experience was assessed in both studies using a user experience questionnaire (post) and changes in psychological variables, in particular pain and mood (pre and post). In Study II, we added an interview with open-ended response format following the intervention to gain further insights on user experience.

### 2.2 Participants

For the feasibility study, a total of 42 participants were recruited across two studies: Study I included *n*_1_ = 20 participants (14 females, 6 males; 24–74 years, M = 37.50, SD = 15.79) and Study II *n*_2_ = 22 participants (13 females, 9 males; 20–67 years, M = 39.63, SD = 15.47). The final sample size in Study I decreased to 18 participants (*n*_1_ = 18) after excluding two individuals due to subclinical rheumatic complaints and migraine symptoms during the intervention period. Inclusion criteria were age of majority, proficiency in German language, and CLBP pain intensity of below 4 on the numerical rating scale (range 0–10), as scores of 4 and above were considered moderate pain (26, 33). As this investigation constitutes a technical feasibility study, it exclusively included participants with CLBP scores below 4 on a 0–10 numerical scale. This criterion was employed to prevent the inclusion of individuals experiencing moderate pain, defined as scores of 4 or higher (26), thereby avoiding the imposition of undue burden on patients with severe pain. CLBP is characterized by a duration of at least three months (1, 34). Exclusion criteria comprised medical and psychotherapeutic conditions (e.g., schizophrenia or epilepsy) and health limitations affecting physical activity. The criteria for inclusion and exclusion were disseminated through recruitment flyers, requested via telephone during the appointment scheduling process, and subsequently verified on-site prior to the commencement of the intervention. The sample size of each study exceeded the minimum of 12 participants recommended for pilot studies (35). No formal sample size calculation was performed, as this study was designed as a pilot.

### 2.3 Measurements

#### 2.3.1 User experience

For user experience evaluation of the psychoeducational intervention in Study I, a 13-item short version of the User Experience Questionnaire [UEQ; (64)] was employed to assess attractiveness, pragmatic quality, and hedonic quality [UEX; (36)]. In Study II, the original 26-item UEQ version (64) was utilized to evaluate the enhanced AR-based psychoeducational prototype with body scan, which distinguishes between attractiveness, pragmatic quality (comprehensibility, efficiency, and reliability), and hedonic quality (stimulation and novelty). The UEQ showed good internal consistency, with Cronbach's alpha ranging from 0.65 to 0.89 (64). Both questionnaires utilized a response scale ranging from −3 (“most negative”) to +3 (“most positive”).

#### 2.3.2 Pain intensity

The Numeric Rating Scale [NRS; (37)] is a validated questionnaire consisting of a single item that assesses the pain intensity (PI) of CLBP on a scale from 0 (“no pain”) to 10 (“worst pain”). The NRS exhibits excellent test-retest reliability, intraclass correlation coefficient and strong convergent validity with the Visual Analog Scale (VAS), with a correlation coefficient of 0.93 (38).

#### 2.3.3 Mood

The short scale for assessing positive activation, negative activation, and valence in experience-sampling studies [PANAVA-KS; (39)] is a bipolar tool for mood evaluation. Valence reflects the hedonic tone of an emotional experience and complements the activation dimensions of positive and negative affect. Participants rated their pre-intervention feelings using two valence items and four items each for negative and positive affect on a 7-point scale ranging from −3 to +3. The interpretation of the scores varied across subscales. Lower (negative values) values of valence and positive affect scores indicate a decline in mood, whereas lower (negative) affect scores indicate less negative emotions, hence better mood. The tool demonstrated high reliability, with Cronbach's alpha ranging from .92 to .94.

#### 2.3.4 Interview questions

In semi-structured interviews, the subjective experiences of participants were captured using a tailored interview guide based on a translated and adapted study of VR applications for patients with CLBP (40) with an open-ended response format. The questions from Smits et al. (40) were translated from English to German and adjusted for the AR application, resulting in the following revised questions: (1) What are your thoughts about AR usage? (2) Where and when do you use AR? (3) Does AR affect your sense of security? (4) How do you rate the comfort of the AR experience? (5) What is your ideal AR experience? (6) Would you use AR as a tool in future inquiries?

### 2.4 Interventions

The development of the interventions for Studies I and II was based on a scoping review (31), which incorporated the Unified Theory of Acceptance and Use of Technology (32) and the Health Action Process Approach (41, 42) for the psychoeducational treatment development in AR. The resulting recommendations pertained to the psychoeducational content, psychological learning factors, technical framework conditions, and outcome variables of pain-psychological interventions for AR as key domains. The interventions of Studies I and II were conducted once and lasted approximately 40 min. AR was used to facilitate knowledge about CLBP. Table 1 outlines the intervention procedure for Study I.

**Table 1:** Study flow of the assessment and psychoeducation intervention tailored for chronic low back pain (CLBP) in augmented reality (AR) using the Microsoft HoloLens 2 ®.

Intervention phase	Interventions content
Pre-phase	Survey of psychological variables such as pain intensity and mood in AR. Socio-demographic variables were collected before the intervention using a paper-pencil questionnaire
Phase 1 Technical Tutorial	Tutorial unit using an agent for practical operating exercises (e.g., holding, pulling): During the intervention, users can use the digital help button for anxiety, technical problems, content-related information or to exit the application, which is available throughout the intervention.
Phase 2 Intervention: Psychoeducation	Start of application with simulation of a peer-to-peer interaction in which a digital agent reports the symptoms of his chronic back pain: User can interact with the agent through yes/no answers.
Phase 3 Intervention: Psychoeducation	Another digital agent (“digital therapist”) appears and offers support. The digital therapist agent presents the user a table that differentiates between acute and chronic back pain. The user is instructed to complete the table in the interactive elements, as shown in Figure 1a.
Phase 4 Intervention: Psychoeducation	Interactive elements help the user to learn about the individual factors of a (a) biological, (b) psychological, and (c) social vicious circle of CLBP.
Phase 5 Intervention: Psychoeducation	An explanatory unit with animations converts the individual vicious circles into gears and the digital therapist agent explains the interplay of (a) biological, (b) psychological, and (c) social factors in CLBP, as shown in Figure 1b.
Phase 6 Intervention: Psychoeducation	Digital therapist agent educates users about treatment options using the bio-psycho-social model.
Phase 7 Intervention End	Farewell by digital therapist agent
Post-phase	Survey of the psychological variables in AR as in pre-phase

The intervention in Study II was extended to address the relationship between stress and CLBP and integrated the Body Scan technique according to Kabat-Zinn (43, 44) for stress reduction in a digital forest landscape. Table 2 delineates the intervention procedure for Study II.

**Table 2:** Psychoeducation in combination with a mindfulness body scan tailored for chronic low back pain (CLBP) in sugmented reality (AR).

Intervention phase	Interventions content
Phase 1 Technical Tutorial	A tutorial unit on Microsoft HoloLens 2 ® operation employs an agent for practical exercises (e.g., holding, pulling) as shown in Figure 2a. During the intervention, user's can press a digital help button for anxiety, technical issues, content-related queries, or to exit the application. Fireflies maintain users' attention throughout the intervention. The user's gaze is directed by a firefly, which lights up in order to highlight important elements in the scene.
Phase 2 Intervention: Psychoeducation	Intervention begins with a simulation of a peer-to-professional interaction, in which the user interacts with the agent by choosing different dialogue options. The bio-psycho-social model is explained to user in an interactive way. Information is provided on the effects of stress, animated by red flashes, on the biological, psychological, and social risk factors of pain chronification as well as on the active influence and effect of mindfulness-based stress reduction (MBSR) interventions, as shown in Figure 2b.
Phase 3 Intervention: Psychoeducation with Focus on MBSR	Introduction, explanation of MBSR, and exercise instructions for the MSBR intervention “body scan”
Phase 4 Intervention: MBSR (Body Scan)	A simulated forest landscape is set up, as shown in Figures 2c,d. A therapeutic digital agent appears and instructs the MBSR intervention body scan.
Phase 5 Intervention End	The therapeutic digital agent expresses gratitude for the user's participation, grants permission, and concludes the intervention and simulated landscape.

**Figure 1:**
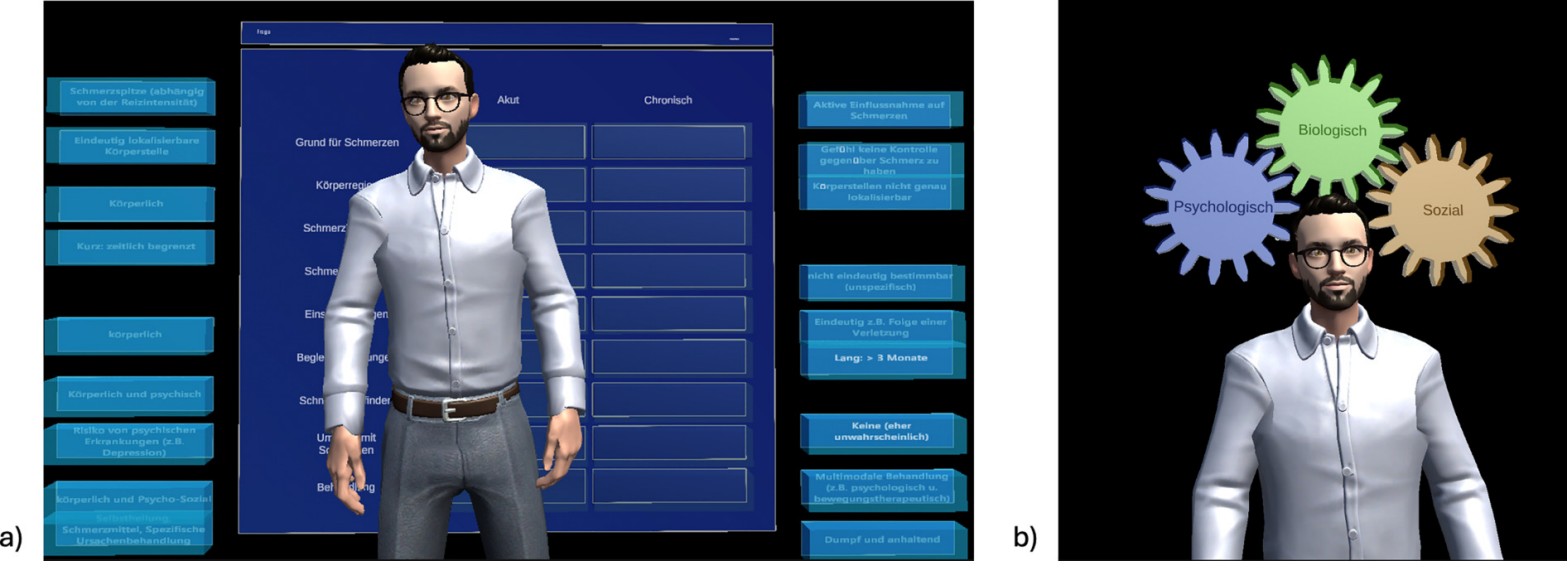
Agent with interactive exercise (table) for differentiating acute vs. chronic back pain **(a)** and explanation of animated bio-psycho-social model **(b)**.

**Figure 2:**
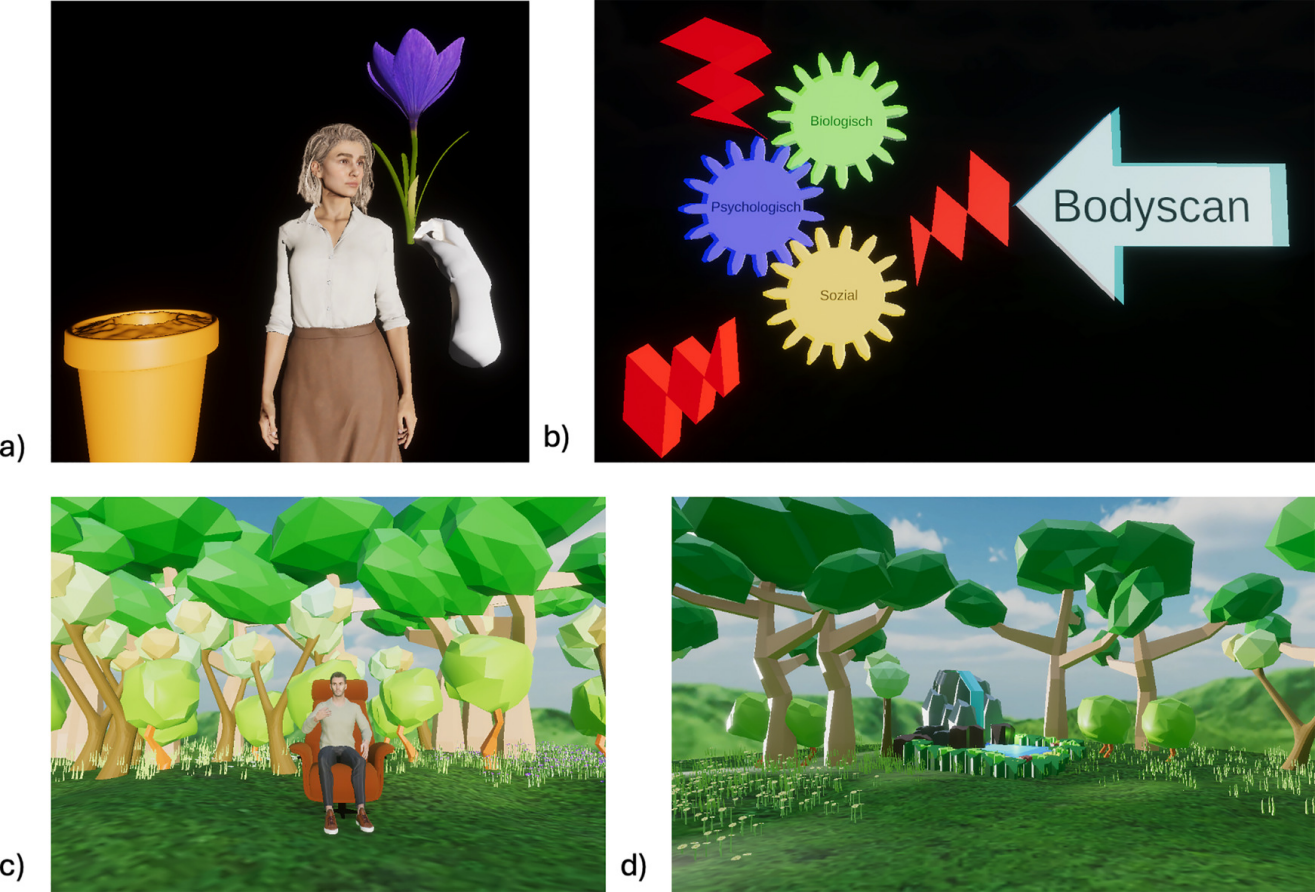
An agent with an interactive module for Microsoft HoloLens 2 ® **(a)** and a psychoeducational tool illustrating the impact of stress on chronic low back pain through a bio-psycho-social framework **(b)**, including a body scan intervention conducted in an augmented reality forest landscape **(c,d)**.

In contrast to Study I, Study II featured an interactive tutorial video that guided users in the operation of the Microsoft HoloLens 2 ®, such as grasping and pulling, prior to the intervention. The tutorial aimed to prevent users from leaving the tracking area during grasping movements and to avoid the snapping back of virtual elements. Additionally, the limited field of view of the HoloLens 2, which made orientation in real space more challenging, was considered in the design. A digital firefly was implemented to direct users' attention toward the positioning of virtual elements in real space, ensuring a seamless intervention process. Figures 1, 2 illustrate the corresponding AR intervention images.

#### 2.4.1 Technical implementation of psychoeducation and MBSR in augmented reality

We used Microsoft HoloLens 2®, whose transparent displays allowed users to see their real environment as if they were not wearing a head-mounted display (HMD). The virtual environment was overlaid and spatially mapped onto the real world, enabling a concurrent experience. The HMD's depth sensors enabled interactions between physical and virtual objects, such as physical objects occluding digital objects or virtual objects interacting with physical surfaces. Reports indicate minimal simulator sickness with the use of the HoloLens (45), which is a notable advantage over VR, in which motion sickness is more prevalent (46, 47). Users interact with virtual objects by tapping or grabbing them with their hands (48). A tutorial scene (Figure 2a) was developed to teach users the necessary hand gestures. The application, developed using Unity 2021.3 and Microsoft's Mixed Reality Toolkit, runs natively on a Windows PC and streams to the HoloLens via the holographic remoting app (49), using the host computer's graphics processing for high-fidelity 3D models and advanced lighting. The investigator was able to view the AR scene from the participants' perspective on the monitor. Users encountered photorealistic characters created and animated with Character Creator 4, featuring blend shapes and rigged bones for facial expressions and realistic lip-syncing. The participants experienced a calming forest environment, with the HoloLens screen adjusted to a high opacity level for enhanced immersion.

### 2.5 Data analysis

The feasibility and user experience of AR interventions tailored for CLBP were quantitatively analyzed using SPSS Statistics version 28.0.1.1 for Windows except for the interview questions. Hypothesis 1 assessed technical feasibility based on dropout rates. Hypothesis 2 regarding user experience of the prototypes was evaluated by comparing the user experience scores in the two studies with benchmark values provided by Schrepp et al. (50). Further aspects of user experience such as change of pain intensity and mood was analyzed in Study I using the Wilcoxon Signed-Rank Test using SPSS's exact test (1-sided), as the due to violation of the normal distribution, and in Study II using paired *t*-tests as normal distribution of the pre-post analysis variables were given. For the statistical tests and interpretation, a significance level of *α* = .05 (1-sided) was employed, the Bonferroni correction was implemented by multiplying the *p*-values with the number of tests conducted (factor 8, due to two tests per variable), and Cohen's d effect sizes (51) were utilized. All reported *p*-values are Bonferroni-corrected unless stated otherwise. User experience data based from semi-structured interviews were analyzed via thematic analyses for each question to explore participants' perceptions and behaviors (52).

## 3 Results

Both samples included 13 master's and 10 bachelor's degree holders, eight secondary, seven middle school, and two basic secondary graduates. Pain duration deviated significantly from a normal distribution in both studies (*p* < .001), with a mean duration of 1.80 months (SD = 8.05) in Study I and 53.90 months (SD = 105.28) in study II. Pre-intervention pain intensity was M = 1.05 (SD = 1.98) in Study I and M = 1.77 (SD = 1.34) in Study II. A detailed overview of the sample statistics is provided in Supplementary Appendix 1.

### 3.1 Feasibility (dropout rate) of augmented reality intervention

The technical feasibility of AR interventions was evaluated based on the dropout rate of less than 50% (H1). In Study 1 (*n*_1_ = 18), 18 out of 20 participants (90%) successfully completed the psychoeducational AR intervention, while two (10%) discontinued the intervention due to migraine and hand pain (H1a). In Study II, all participants (*n*_2_ = 22) successfully completed the AR intervention, resulting in a dropout rate of 0% (H1b). These results rendered support for hypothesis 1 postulating the technical feasibility of an AR intervention adapted for CLBP, in particular a psychoeducational intervention (H1a) and psychoeducation combined with a MBSR-based body scan (H1b).

In Study I, the technical feasibility of administering questionnaires in AR was also assessed. The low attrition rate suggests that questionnaires can be administered in the AR environment and were deemed acceptable by the study participants. However, participants were often frustrated with the handtracking of the hololens during the AR-questionaire. Therefore, we decided to use the pencil-and-paper questionnaire for the second study. Psychometric analysis of the scales showed similar results to conventional assessments. Scale statistic (e.g., internal consistency) are presented in Supplementary Appendix 2.

### 3.2 User experience of augmented reality intervention

**Figure 3:**
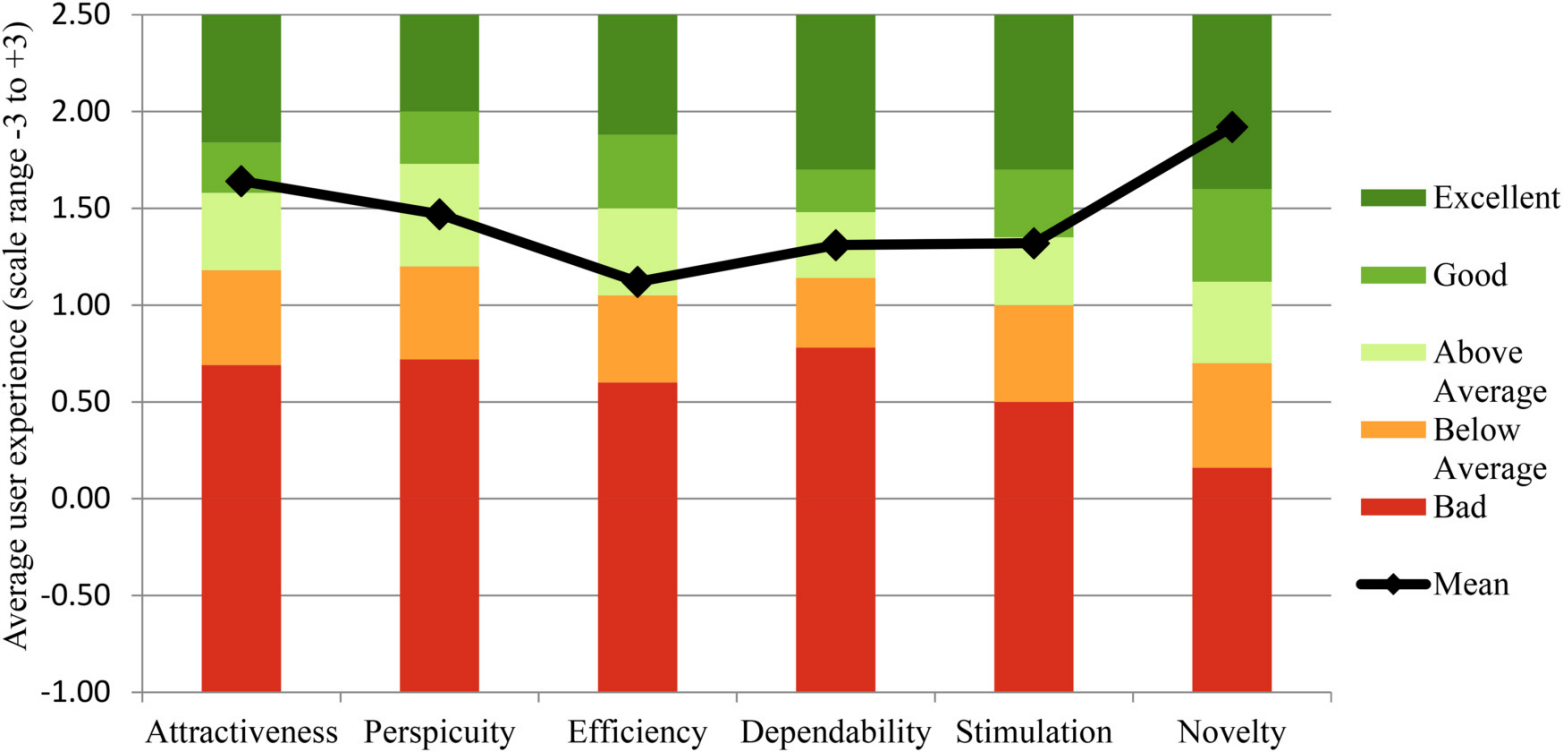
User experience questionnaire dimensions of the augmented reality prototype in study II for pain-specific psychoeducation combined with a body scan for chronic low back pain (black dots) compared with the benchmark (bars) by Schrepp et al. (50).

Hypothesis 2 examined the user experience of the psychoeducational AR intervention (H2a, Study I) and the user experience of the advanced psychoeducational AR intervention combined with an AR body scan (H2b, Study II). The comparison analysis based on a UEQ benchmark with 246 product evaluations (50) revealed that reported user experience evaluation of Study I and Study II achieved acceptable “Good” ratings in the overall assessment of attractiveness. Regarding pragmatic quality, ratings improved from “Below average” in Study I to “Above average” in Study II. Both studies achieved an “Excellent” rating in hedonic quality. Table 3 and Figure 3 present the detailed user experience values for both studies and graphical representations for Study II. Overall, our findings confirm hypothesis 2 (H2a and H2b). Additionally, the enhanced application in Study II (psychoeducation and body scan) showed improvements in user experience, particularly in novelty and attractiveness.

**Table 3:** Scale statistics of the dimensions of user experience for the evaluated augmented reality prototype of Study I and Study II and their evaluation referring to benchmarks provided by Schrepp et al. (50).

Dimension Study I	Dimension Study II	Mean	SD	Comparison to benchmark	Interpretation
Attractiveness		1.54	0.88	Good	10% of results better, 75% of results worse
	Attractiveness	1.64	0.88	Good	10% of results better, 75% of results worse
Pragmatic Quality		1.05	1.01	Below Average	50% of results better, 25% of results worse
	Perspicuity	1.47	1.30	Above Average	25% of results better, 50% of results worse
	Efficiency	1.12	0.87	Above Average	25% of results better, 50% of results worse
	Dependability	1.31	0.99	Above Average	25% of results better, 50% of results worse
Hedonic Quality		1.70	1.06	Excellent	In the range of the 10% best results
	Stimulation	1.13	0.91	Above Average	25% of results better, 50% of results worse
	Novelty	1.92	0.84	Excellent	In the range of the 10% best results

We further analyzed pain intensity and mood to explore additional facets of user experience and evaluate whether an AR intervention, either as an psychoeducational intervention for CLBP (Study I) or in combination with a body scan (Study II), can improve these pain-related psychological variables. Study I showed no significant changes in pain (*p* > .05) and no improvement in all facets of mood (*p* > .05). Study II demonstrated a significant reduction in pain (*p* = .004) with medium effect size. Further, a significant decrease in negative affect (*p* = .003) and a change in valence (*p* = . 026), both with medium effect sizes, were observed (Table 4). Overall, the analysis results on pain intensity and mood underline the good user experience of the AR prototypes as no worsening or even significant enhancement in emotional variables were reported.

**Table 4:** Pain intensity and mood changes pre- and post-augmented reality intervention.

	Scales	Pre Intervention I: Mdn (IQR) II: Mean (SD)	Post Intervention I: Mdn (IQR) II: Mean (SD)	Test value I: z II: T	Wilcoxon-test: *n*−; *n*+; (W-;W+); Number o f bonds	*p* value BoC	ES d
Study I	Pain Intensity	0.00 (1.00)	0.00 (0.00)	−1.60	3; 0 (6;0) 15	>0.999	−0.82
Study II		1.77 (1.34)	0.95 (1.04)	3.81		0.004**	−0.81
Study I	Valence	2.00 (1.00)	2.00 (1.13)	−0.37	7; 4 (37;29) 7	>0.999	−0.17
Study II		1.40 (.98)	1.86 (.91)	−3.02		0.026*	0.64
Study I	Positive affect	0.75 (1.63)	0.50 (2.31)	−2.32	13; 2 (100;20) 3	0.064	−1.30
Study II		0.79 (1.13)	0.72 (1.13)	0.23		3.282	−0.05
Study I	Negative affect	−1.75 (1.81)	−1.75 (1.75)	−0.47	6, 4 (32;23) 8	>0.999	−0.22
Study II		−1.15 (1.00)	−1.94 (.89)	3.91		0.003**	−0.83

Study I, intervention psychoeducation for CLBP; Study II, intervention psychoeducation for CLBP in combination with body scan; IQR, inter quartile range; BoC, bonferroni correction; *n*−, negative rank; *n*+, positive rank; W−; W+, rank sum of negative and positive differences; ES, effect size (Cohen's d).

* *p* < .05 (1-sided).

** *p* < .01 (1-sided).

### 3.3 Exploratory analyses (qualitative participant feedback)

The semi-structured interview results corroborate our quantitative findings on user experience, offering further insights into enhancing the usability and user experience of the AR prototype. A summary of the responses is provided in Supplementary Appendix 3.

Question 1. Twenty participants rated the AR experience positively with comments such as “P3: Exciting. Use of (resources) when no person is present” and “P12: I would be thrilled if AR could be integrated into the therapeutic context.” Two participants suggested improvements, such as a better field of view for Microsoft HoloLens 2® or a more human voice.

Question 2. Of the participants, 14 had no previous experience with AR, four had used it in studies, and two had used it in games. Two participants had experience with body scan units, one in yoga and one in psychotherapy. Inexperienced users showed a reduced sense of security and preferred realistic representations, whereas experienced users suggested technical improvements and were more inclined to reuse the AR prototype.

Question 3. Nineteen participants stated that the AR had no effect on their sense of safety. One participant reported uncertainty due to the limited field of view, and another due to the problematic hand tracking of the Microsoft HoloLens 2®. Another study expressed uncertainty regarding hologram alignment. Additional feedback from Study I pointed to the suboptimal hand tracking capabilities of HoloLens 2®. The participants criticized the size of the device, the limited field of view, and discomfort. Users have difficulty grasping virtual objects because their movements often go beyond the tracking area, causing the object to reset.

Question 4. During the AR experience, 15 participants had different perceptions of comfort, e.g., “P05: The glasses were more noticeable,” “P09: Uncomfortable head area during body scan,” and “P18: Comfortable and uncomplicated.”

Question 5. For an “ideal AR experience,” five participants wanted more realistic graphics. Two requested better audio quality for the agent voices and two wanted background music during the body scan. Three test subjects wanted lighter glasses, e.g., “P09: As if you're not wearing anything.” Seven participants were satisfied with the prototypes.

Question 6. Fourteen participants used AR for future questions. One would only use it when ill instead of visiting a doctor, and three rejected it because of problematic hand tracking with Microsoft HoloLens 2®.

## 4 Discussion

The primary aim of this study was to evaluate the feasibility and user experience of two interventions tailored for CLBP in two single-armed studies: Study I (Psychoeducation) and Study II (Psychoeducation + Body Scan) in AR. The development of both interventions was based on the results of a scoping review (31) on designing theory-based AR psychoeducational interventions for CLBP. Our study contributes to the empirical understanding of user experience in an AR based psychoeducational and relaxation intervention for CLBP.

### 4.1 Feasibility of augmented reality intervention tailored to CLBP

We investigated the feasibility of AR interventions, specifically an psychoeducational intervention tailored for CLBP and a pain-specific psychoeducational intervention combined with MBSR-Body Scan. The attrition rate in Study I for psychoeducation was 10%, whereas the advanced psychoeducational AR intervention combined with mindfulness-based exercise for patients with CLBP had an attrition rate of 0%. Our findings on AR applications align with existing literature, which supports the feasibility of other head-mounted applications such as VR for the treatment of CLBP. For instance, a study on TBed VR game system for chronic non-specific lower back pain also reported a 0% dropout rate, indicating high feasibility (53). Furthermore, our results are consistent with a multiple experimental single-case study with eight participants for pain psychoeducation and management of CLBP in VR with a dropout rate of 0% (26). A randomized control pilot study of 22 participants investigated the use of VR exergames as a supplement to multimodal pain therapy for CBP in older adults also reported a dropout rate of 0%. Our AR intervention is technically comparable to existing VR treatments, and offers an alternative approach for treating CLBP.

Furthermore, it is important to consider the low pain intensity of the samples and its potential impact on the dropout rate. A randomized study investigating the feasibility of VR for neuroscientific pain education (VR-PNE) in patients with CLBP reported adherence rates of 63.6% for VR-PNE and 63.2% for physical therapy. Concurrently, the sample size, elevated pain scores prior to the study, and a substantial dropout rate (with only 32 out of 52 participants completing the study) indicate that pain intensity may affect the dropout rate, despite this factor not being explicitly examined (54). This finding is corroborated by a correlation between sensitization and pain intensity in CLBP patients (55). Higher pain intensity in CLBP is associated with increased central sensitization, which may influence dropout rates and should be taken into account in future feasibility studies involving patients with higher pain intensity in CLBP.

Future studies should further investigate its technical viability in clinical populations with more severe CLBP. Additionally, in Study I, data collection via questionnaires in AR also proved to be feasible in general, demonstrating acceptable psychometric properties and confirming their practicability, user-friendliness, and reliability. However, we advise against lengthy questionnaires in AR, as the problematic tracking may lead to prolonged application duration and potential frustration. Similar findings were reported for research on questionnaire integration into VR applications (56). Two participants did not complete the study due to pre-exisiting hand pain and subclinical migraine symptoms in Study I. Therefore, we recommend considering these symptoms when defining the inclusion criteria for study participants in AR using current technology, especially if the user is required to do many hand gestures. These kind of problems might not be relevant anymore for devices with optimized technology. Such issues may become irrelevant with advancements in AR technology.

### 4.2 User experience of augmented reality intervention tailored to CLBP

The optimized AR prototype from Study II, which integrated psychoeducation with a MBSR body scan, resulted in above-average to excellent user experiences, an improvement in mood, in terms of less negative emotions. Our findings on AR interventions align with the high acceptance and satisfaction achieved by VR applications, such as VR-based pain neuroscience education among CLBP patients, with a virtual reality application of pain neuroscience education (VR-PNE) achieving higher satisfaction scores than conventional physiotherapy (54). Our results were also consistent with a randomized control pilot study on VR exergames as an adjunct to multimodal pain treatment in older adults with chronic back pain, where attractiveness and perspicuity were rated as “very good,” efficiency, dependability, and stimulation as “good,” and novelty as “above average” (57). Compared to the pilot study by Stamm et al. (57) on VR exergames as an adjunct to multimodal pain management in older adults with chronic back pain, our prototype performed less favorably across all user experience scales, with the exception of the “novelty” dimension, which was rated significantly higher in our study.

Our results from Studies I and II demonstrate that AR intervention does not exacerbate pain intensity in mild pain conditions. This corresponds to a randomized control study on pain psychoeducation and pain management in VR for CLBP, which found non-clinically relevant pain reduction in some patients (26). In contrast to Study I, Study II demonstrated an reduction in pain intensity through the integration of psychoeducation and the MBSR body scan. These findings align with existing research on conventional MBSR therapies, which indicate that body scan techniques exert beneficial effects on CLBP and enhance quality of life (58, 59). It is important to note that in Study I, there was minimal variation in baseline pain levels in terms of a floor effect in the distribution of the average pain level of the participants, which posed challenges in demonstrating the effects of pain reduction. Conversely, Study II exhibited greater variability in pain pre- and post intervention, thereby facilitating the detection of pain reduction effects. The distracting effect of VR through neuromodulation and graded exposure therapy (23) has been shown to be particularly effective, as it shifts patients' attention away from pain and significantly reduces pain intensity and interference (60, 61). Similar effects may also apply to our AR interventions; however, this requires further investigation in future studies.

Furthermore, Study II demonstrated a significant reduction in negative affect following mindfulness-based body scan intervention in an AR forest landscape, which is consistent with VR studies showing improved mood and well-being in simulated natural environments as well as increased immersion. For instance, older adults appeared to benefit from simpler virtual environments, emphasizing the importance of the intervention design (62). These results suggest the potential for further AR studies using simulated landscapes in health contexts.

However, in our AR application, the user experience received lower ratings in the user experience subscales compared to some VR studies with exergames for CBP (57). One possible explanation is distraction caused by tactile, visual, and auditory stimuli in VR with higher immersion. Therefore, we recommend that future research on AR applications should address these stimuli and investigate the impact of immersion intensity on distraction and user experience. Furthermore, some users' discomfort leading to lower scores on user experience in our AR study may be attributed to the use of HoloLens 2 ®. The poor user experience rating in Study I could be attributed to the use of the HoloLens 2 ®, too. In the advanced prototype of Study II, this problem was addressed by tutorial exercises on technical operation and attention guidance, which led to significantly better ratings. The below-average hand tracking of HoloLens 2 ® requires additional training exercises and an interactive tutorial. Several participants expressed frustration during the intervention or reported that the HoloLens 2 ® was too large, had a limited field of view, and caused discomfort.

Specifically, participants encountered difficulties in grasping and manipulating virtual objects, as they focused on the interaction target rather than the virtual object itself, resulting in their departure from the tracking area and causing the virtual object to revert to its original position. Consequently, the HoloLens ® prematurely terminated the movement. This mechanism became a source of frustration. In this context, the lack of change in mood in Study I and subsequent improvement in Study II can be attributed to the enhanced prototype in Study II, which facilitated more intuitive operation. A study on the development of usability heuristics for AR and VR underscores the importance of user-friendly interfaces to mitigate issues such as information overload (63). To prevent users from moving objects beyond their reach, future applications should offer alternative modes to enhance reality perception and optimize processes. We recommend for future HoloLens ® applications, particularly for inexperienced users, to rely less on grasping and moving gestures and instead prioritize the more reliable tapping gesture. Lastly, our results from Study I showed a descriptive, albeit non-significant decline in positive affect which may be attributed to limitations in the hand tracking systems, highlighting the technical prerequisites for ensuring the usability of AR interventions. Overall, our AR interventions adapted for CLBP offered a positive user experience, including the enhancement of negative mood and pain. At the same time, our study highlighted fields of technical and design improvement to optimize the user experience.

### 4.3 Limitation

Concurrently, it is necessary to acknowledge the limitations of this study: First, two separate AR intervention studies without a control group were compared with conventional intervention. Second, the small sample size limits statistical power and reduces the identification of significant effects. Third, generalizability to clinical applications are limited due to selection bias, as participation was likely influenced by high motivation among individuals. Fourth, the lack of blinding of participants and study conductors may have lead to a bias in user experience results due to the Hawthorne effect. Fifth, there may be a potential bias resulting from the recruitment of participants who have a specific interest in AR.

## 5 Conclusion

This study investigated a psychoeducational AR intervention and in combination with a mindfulness-based body scan for individuals with CLBP symptoms. The empirical results demonstrated the technical feasibility of psychoeducational AR interventions, practicability of AR questionnaires, and good overall user experience. This study provided valuable insights for future studies on design improvements from a technological and psychological perspective to enhance user experience. In summary, the tested AR prototypes represent promising psychoeducational tools for patients with CLBP, aligning with treatment guidelines and laying the groundwork for further clinical research.

## Data Availability

The raw data supporting the conclusions of this article will be made available by the authors upon reasonable request.
