# Supplementary material for: Feasibility and user experience of augmented reality psychoeducation and mindfulness body scan for chronic low back pain
Source: Front Pain Res (Lausanne). 2025 Jul 1;6:1600637. doi: 10.3389/fpain.2025.1600637 (PMC12281825; doi:10.3389/fpain.2025.1600637)
Supplement: Supplementary file 1 [file Table4.docx]

**Appendix** 2. Psychometric analysis of questionnaires administered in augmented reality

| Construct: Subscale | N | Item  Count | $\alpha$ | Discrimination  Range (Min,Max) |
| --- | --- | --- | --- | --- |
| Mood: Valence | 19 | 2 | .796 | .688 |
| Mood: Positive affect | 19 | 4 | .668 | .573 - .644 |
| Mood: Negative affect | 19 | 4 | .795 | .681 - .785 |
| UE: Attractivness | 18 | 4 | .862 | .794 - .851 |
| UE: Pragmatic Quality | 18 | 4 | .789 | .669 - .803 |
| UE: Hedonic Quality | 18 | 3 | .583 | .300 - .585 |

Note: N = N of Study I, mood from the pre-survey and user experience (UE) from the post-survey,

UE = User Experience, $\alpha$ = Cronbach’s alpha
