# Supplementary material for: Feasibility and user experience of augmented reality psychoeducation and mindfulness body scan for chronic low back pain
Source: Front Pain Res (Lausanne). 2025 Jul 1;6:1600637. doi: 10.3389/fpain.2025.1600637 (PMC12281825; doi:10.3389/fpain.2025.1600637)
Supplement: Supplementary file 2 [file Table5.docx]

**Appendix 3.** Detailed results oft he exploratory anaylsis of study II (participant feedback)

| **Interviewquestion** | **Answer** |
| --- | --- |
| (1) What are your thoughts  about AR usage? | Question 1. Twenty participants rated AR usage positively, as illustrated by comments such as "P3: Exciting. Use (resources) when no person is present. Easy to use without prior knowledge” and "P12: I would be thrilled if AR could be integrated into the therapeutic context". Two participants suggested improvements, such as a better field of vision for Microsoft HoloLens 2 ®, holograms without glasses, or a more human agent’s voice. |
| (2) Where and when do you  use AR? | Regarding prior AR usage, 14 participants had never used AR, 4 had used it in studies, and 2 had used it in games like "PokemonGo" or on rollercoaster rides. Additionally, two participants had prior experience with body scan units in therapeutic settings - one in the context of yoga exercises and another for treating attention deficit hyperactivity disorder in psychotherapy. Furthermore, our findings indicated that inexperienced users experienced a slight impairment in their sense of security, which was not reported by experienced users. Inexperienced users demonstrated a preference for realistic representations for an optimal AR experience, whereas experienced users emphasized technical improvements in their feedback. Experienced users exhibited a tendency to reuse the AR prototype, while inexperienced users expressed a preference for conventional methods. |
| (3) Does AR affect your  sense of security? | Concerning the impact of AR on participants’ sense of security, 19 participants reported no effect. One participant reported feeling insecure due to the limited field of view, while another felt uncertain regarding the initial operation and problematic hand tracking of the Microsoft HoloLens 2 ®, which sometimes only registered hand movements after multiple repetitions. Another participant expressed mild uncertainty about the alignment of holograms and whether movement towards the holograms within the room was permissible. |
| (4) How do you rate the  comfort of the AR  experience? | Question 4. Regarding the comfort of the AR experience, 15 subjects found it pleasant, e.g., “P05: The glasses became more noticeable over time.", "P09: The head region felt uncomfortable during the body scan", "P10: Difficulties with balance due to heavy glasses" and "P18: Comfortable to wear and uncomplicated." |
| (5) What is your ideal AR  experience? | Question 5. For the "ideal AR experience," five participants wanted more realistic graphics. Two of them suggested improving the audio quality by making the agents' voices sound more realistic and two wanted background music during the body scan. Three subjects preferred lighter glasses for relaxation interventions, e.g., "P06: Glasses like those from an optician or diving" or "P09: If you couldn't feel the glasses, as if you weren't wearing anything." Seven subjects were satisfied with the prototypes without any changes, e.g., "P18: I was satisfied as it was." |
| (6) Would you use AR as a  tool in future inquiries? | Question 6. When asked about the use of AR in answering future questions, 14 participants responded affirmatively. One would use it only in cases of illness to avoid doctor visits, and three rejected the idea because of problematic hand tracking using Microsoft HoloLens 2 ®. |
